# A Switch to Polyunsaturated Fatty Acid Based Ketogenic Diet Improves Seizure Control in Patients with Drug-resistant Epilepsy on the Mixed Fat Ketogenic Diet: A Retrospective Open Label Trial

**DOI:** 10.7759/cureus.6399

**Published:** 2019-12-16

**Authors:** Janak Nathan, Sonal Bailur, Kuldeep Datay, Suvasini Sharma, Dhanashri Khedekar Kale

**Affiliations:** 1 Neurology, Shushrusha Hospital, Mumbai, IND; 2 Clinical Nutrition & Dietetics, Dr. Nathan Sanjiv Clinic, Mumbai, IND; 3 Statistics, Dr. Nathan Sanjiv Clinic, Mumbai, IND; 4 Pediatrics, Lady Hardinge Medical College, New Delhi, IND

**Keywords:** drug-resistant seizures, omega-3 fatty acids, omega-6 fatty acids, docosahexenoic acid, ketogenic diet

## Abstract

Introduction

The ketogenic diet (KD) is used for drug-resistant epilepsy. However, some patients find only a modest benefit, which may plateau over time. Evidence from several animal and human studies suggests that polyunsaturated fatty acids (PUFAs) may be a beneficial form of treatment for these patients. This retrospective study was conducted to evaluate whether a switch from classic mixed fats KD (MFKD) to a natural polyunsaturated fatty acid KD (PUFA-KD) would improve seizure control.

Methods

The study evaluated the medical paper record forms of patients who had at least one seizure per week despite the use of MFKD. These patients were started on PUFA-KD and grouped according to the oils preferred. We analyzed the effect on seizure control, tolerability, blood lipids, and adverse effects and whether the type of seizures, age of seizure onset, age at which KD was started, and the ratio of omega 6: omega 3 (n6:n3) fatty acids had any effect on seizure control.

Results

Data from fifty patients (aged 10 months to 35 years) were analyzed. At the end of six and 12 months on the PUFA-KD, 12% (6) and 16% (8) were seizure-free and 82% (41) and 88% (44) had a >50% reduction in seizures, respectively. The mean seizure control at 12 months was highest in patients with mixed seizures followed by those with generalized seizures and lowest for those with focal seizures. Seizure control at 12 months was inversely correlated to the age of onset of epilepsy and age at initiation of KD. This improvement was independent of the type of PUFAs and the ratio of n6:n3 used. The PUFA-KD was generally well tolerated. Blood lipid levels significantly improved.

Conclusion

Changing to PUFA-KD improved seizure control in patients who did not respond satisfactorily to MFKD.

## Introduction

Ketogenic diet (KD) is a high-fat, low carbohydrate and normal protein diet, which is useful in the treatment of drug-resistant epilepsy. Across studies, it has been noted that 50% of patients on KD have >50% reduction in seizures and 10-20% become seizure-free [1]. It is a frequent clinical observation (personal experience and anecdotal) that after the first few months on KD, some patients experience a plateau in seizure reduction, and therefore, other therapies need to be considered. Often such patients wish to continue KD because of the seizure reduction and other benefits such as improved alertness, cognition, and behavior.

Although there has been much research in the last two decades on the clinical utility of the ketogenic and other similar diets, such as the modified Atkins and the low glycemic index diet, there has been little emphasis on the type of fat used in the diet except for the medium-chain triglyceride KD. Traditionally saturated fats have been the prominent fat used [1]. There is limited clinical data on the use of polyunsaturated fatty acids (PUFAs) for the treatment of epilepsy. In vitro and in vivo studies have demonstrated the anticonvulsant action of PUFAs [2]. One of the possible mechanisms of action of KD could be related to omega-3 PUFAs which have been reported to be elevated in the plasma of children on the KD [3]. In vivo studies suggest that this is possibly due to a selective mobilization of PUFAs from adipose tissue or liver, with subsequent incorporation into the brain [4].

There have been a few clinical studies on the use of PUFAs (mostly as omega-3 PUFAs supplements) in epilepsy with conflicting results [5-8]. Also, there is a paucity of data on the type of PUFAs used, namely, omega-3 (n-3), omega-6 (n-6) fatty acids or docosahexenoic acid (DHA), and the optimum n6:n3 ratio which might be effective in seizure control. We hypothesized that combining PUFAs with KD, i.e. using natural PUFAs rich oils as the fat source in KD might improve the anti-seizure efficacy of the KD. Therefore, this retrospective study was conducted to evaluate the effect of a change to PUFA-KD in those on MFKD but with a plateau in seizure reduction. Seizure reduction, tolerability, and blood lipid levels were analyzed after the change to PUFA-KD and whether the n6:n3 ratio had any effect on these parameters.

## Materials and methods

This retrospective, open-label study was conducted from April 2011 to March 2018 in a specialty ketogenic diet clinic in Mumbai, India. Persons who had reached a plateau in their seizure control with the MFKD, that is, the seizure frequency was at least one per week after a trial of MFKD for at least 3 months, were selected. The MFKD in our center is administered as a mixture of saturated, mono-unsaturated fatty acids (MUFA) and PUFAs [9]. This consists of ghee (clarified butter), butter and cream, groundnut or peanut oil and sunflower oil, delivering 25% saturated fats, 37.5 % MUFA and 37.5% PUFAs (21.37%, n-6 and 0%, n-3).

The patients were administered PUFA-KD, using the same KD ratio and calories as MFKD, but the fat used was PUFAs rich oils, namely, safflower oil, flaxseed oil, and sea cod oil. Safflower oil contains only n-6 PUFAs (linoleic acid). Flaxseed oil contains both n-3 (alpha-linolenic acid) and n-6 (linoleic acid) PUFAs. Sea cod oil contains only n-3 PUFAs eicosapentaenoic acid (EPA) and DHA. The composition of these PUFAs oils used is shown in (Table 1). 

**Table 1:** Composition of the PUFA and other fatty acids in PUFA ketogenic diet ALA, α-linolenic acid; LA, Linoleic acid; EPA, eicosapentenoic acid; DHA, docosahexenoic acid; 16:1∆9,  palmitoleic acid; 18:1∆9, oleic acid; C16, palmitic acid; C18, stearic acid; C20, arachidic acid; PUFA, polyunsaturated fatty acid; MUFA, monounsaturated fatty acid; SFA, saturated fatty acid

SOURCE	PUFA		MUFA		SFA		
	N-3 (ALA)	N-6 (LA)	16:1∆^9^	18:1∆^9^	C16	C18	C20
Safflower Oil	-	73.50%	-	16.7%	7.80%	2.10%	0.80%
Flaxseed Oil	55.20%	17.00%	-	19%	6%	2.5%	0.5%
Sea Cod Liver Oil	EPA 6.6% DHA 6.10%	-	-	-	-	-	-

Vegetarians did not agree to use sea-cod oil and hence used either safflower and flaxseed oil or only safflower oil as some vegetarians also refused flaxseed oil due to its taste. Accordingly, there were three groups of patients: Group 1 who had safflower oil (54 ml-180 ml/ day), Group 2 who had safflower (20 ml-100 ml/day) and flaxseed oil (10 ml-50 ml/day), and Group 3 who had safflower (40 ml-180 ml/day), flaxseed (10 ml-30 ml/day) and sea cod oil (20 ml-50 ml/day). The proportion of the oils used in group 2 and 3 were varied, as per tolerability and affordability. The proportion of the oils was specified and calculated by our dietitian team and given to the patients. Calories and KD ratio were maintained at the same level during PUFA-KD as on MFKD.

A daily seizure diary was maintained before the introduction of the MFKD and during the entire period of the MFKD and the PUFA-KD. The seizure frequency was noted at baseline three, six, and 12 months after starting the PUFA-KD. The primary outcome variable was the seizure reduction from the baseline after six and 12 months on the diet defined as responders (those who had >50% reduction in seizures). Tolerability and adverse effects were also documented. In the responders in groups 2 and 3, we also calculated the n6:n3 PUFAs ratio as compared to the non-responders at six months on the diet. Additionally, we also calculated the amount of DHA intake in the diet of the responders in Group 3, as compared to the non-responders at six months on the diet.

Baseline biochemical tests were carried out which included the following: complete blood count (hemoglobin, packed cell volume, mean corpuscular hemoglobin concentration, white blood cells, platelet count) lipid profile (total cholesterol, triglycerides, low-density lipoprotein cholesterol (LDL), high-density lipoprotein cholesterol (HDL), cholesterol/HDL ratio with apolipoprotein A1 & B), fasting sugar, liver function test (serum aspartate aminotransaminase (SGOT), serum alanine aminotransferase (SGPT) blood gases (partial pressure of oxygen, carbon dioxide, and bicarbonate), serum creatinine, serum calcium, total protein, serum albumin, serum globulin, albumin/globulin ratio, serum sodium, serum potassium, serum chloride and urinary spot calcium to creatinine ratio. These were repeated at intervals of three, six, and 12 months. Urine ketone levels were recorded at least three times daily. The effect of blood lipids was assessed of the three groups between baseline, MFKD, and PUFA-KD.

Statistical analysis was performed using SPSS software and one-way repeated measures ANOVA to determine if there is significant seizure control on PUFA KD analyzing baseline, six and 12 months data of 50 subjects. Univariate analysis of variance was used to correlate the type of seizures, age of seizure onset, and age at which KD was started with seizure control at the end of 12 months. A paired-samples *t*-test was conducted to compare the blood lipid parameters of the three groups between baseline, MFKD, and PUFA-KD. Data were found to be statistically significant, when the *p*-value was <0.05.

Institutional ethics committee approval was obtained for the retrospective data analysis of the subjects. Written informed consent was taken from the patients and care-givers.

## Results

There were four adults, seven adolescents, 26 children and 13 infants in the study population of which 39 were male and 11 were female. The duration of the MFKD ranged from 5 to 87 months (M = 23 months). They were on a median of three AEDs at the time of PUFA-KD initiation. There were 11 patients in Group 1, seven patients in Group 2, and 32 patients in Group 3.

Withdrawal from the PUFA-KD dietary treatment was seen in four patients and was due to non-compliance in two patients, non-compliance from the care-givers in one patient, and unsatisfactory seizure control in one patient. Non-compliance was due to the restrictiveness of the diet and the taste and odour of the fish oil. All four patients withdrew completely from KD. All patients maintained high urine ketones (>160 mg/dL) most of the period. 

 At six months on the PUFA-KD, 12% (six) were seizure free and 82% (41) had a >50% reduction in seizures (including seizure free). At 12 months, 16% (8) were seizure free, and 88% (44) had >50% reduction in seizures (including seizure free; Table 2).

**Table 2:** Seizure control on diet N, sample size

	6 months	12 months
Total study population (n=50)	N=50	N=50
Seizure free (100%)	6	8
50-99.9%	35	36
<50%	9	3
No change in Seizures	0	1
Seizures increased	0	2
Group 1 (n=7)	N=7	N=7
Seizure free (100%)	1	2
50-99.9%	6	5
<50%	0	0
No change in seizures	0	0
Seizures increased	0	0
Group 2 (n = 11)	N=11	N=11
Seizure free (100%)	1	0
50-99.9%	9	10
<50%	1	0
No change in seizures	0	0
Seizures increased	0	1
Group 3 (n = 32)	N=32	N=32
Seizure free (100%)	4	6
50-99.9%	20	21
<50%	8	3
No change in seizures	0	1
Seizures increased	0	1

Results show that on an average, the number of seizures per month reduced from a mean of 317.54 (SD = 542.834) on MFKD to a mean of 124.88 (SD = 252.493) on PUFA. Results of the univariate analysis of variance showed mean seizure control at 12 months was highest in those with mixed seizures (M = 76.583, SD = 28.19064) followed by those with primary generalized (M = 67.2120 SD= 49.01852) and then for those with focal onset and secondary generalized seizures (M = 51.8538, SD = 110.98445). These differences between means were statistically not significant (F = 0.435, *p* = 0.650). Seizure control at 12 months was inversely correlated to the age of onset of epilepsy (*r* = -0.256) and also to age at initiation of KD (*r* = -0.259). The later the age of onset and later the age at initiation of KD, the lower the seizure control. However, these correlations were statistically non-significant (*p* = 0.073, *p* = 0.069, respectively). The results of the one-way repeated measures ANOVA showed that there was a significant main effect of change in the type of diet on the average number of seizures per month (F(1,48) = 4.420, *p* = 0.041). Results further showed that in the group of participants with more than 90% improvement on MFKD, the average number of seizures per month reduced from a mean of 90.65 (SD = 213.296) to a mean of 39.94 (SD = 62.222) when the diet was changed to PUFA - a 44.06% reduction. In the group of participants with less than 90% improvement on MFKD, the average number of seizures per month reduced from a mean of 434.42 (SD = 622.115) while on MFKD to a mean of 168.64 (SD = 299.798) when the diet was changed to PUFA - a 38.82% reduction. The difference in seizure reduction between these two groups with PUFA (more than 90% seizure reduction and less than 90% seizure reduction) as compared to the seizure reduction on MOKD was found to be significant (F(1, 48) = 6.043, *p* = 0.018). The patients were still on a median of three AEDs even after 12 months on PUFA-KD.

The average n6:n3 ratio in Group 2 was 4:1 and that in Group 3 was 12.6: 1. The n6:n3 ratio of responders (patients with >50% seizure control) in Group 2 was 4:1 as compared to 4.7:1 in the non-responders. In group 3 patients, the n6:n3 ratio was 14:1 in responders and 14.2:1 in non-responders. The mean DHA intake in Group 3 was 2.2 grams per day. The daily intake of DHA in group 3 in the responders was 2.3 g per day versus 2.1 grams per day in the non-responders. These differences were not statistically significant.

Overall, the PUFA-KD was tolerated well. The main adverse effects included nausea and constipation (14%), which were also seen when they were on MFKD (Table 3).

**Table 3:** Adverse effects on polyunsaturated fatty acid-based ketogenic diet N, sample size

Adverse effect	N (%) (Total N = 50)
Constipation	7 (14%)
Nausea	7 (14%)
Lethargy	3 (6%)
Vomiting	2 (4%)
Diarrhoea	1 (2%)

The mean values of various lipid fractions were compared between the baseline to that on MFKD, baseline to that on PUFA-KD and MFKD to that on PUFA-KD (Table 4).

**Table 4:** Blood lipids profile of patients MFKD, mixed oil ketogenic diet; PUFA-KD, polyunsaturated fatty acids ketogenic diet; M, mean; TG, triglyceride; Chol, cholesterol; HDL, high density lipoprotein cholesterol; LDL, low density lipoprotein cholesterol; Apo A1, apolipoprotein A1; Apo B, apolipoprotein B

	Group 1			Group 2			Group 3		
	Baseline	MFKD	PUFA-KD	Baseline	MFKD	PUFA-KD	Baseline	MFKD	PUFA-KD
TG mg/dl	54-143 (M=85.6)	77-150 (M=108.5)	70.8-191 (M=117.5)	68-231 (M=113.6)	70-147 (M=103)	57-136 (M=84.8)	51-282 (M=102.9)	54-558 (M=128)	52-802 (M=156.6)
Chol mg/dl	109-199 (M=160.2)	147-239 (M=189.7)	119-233 (M=159.8)	112-235 (M=162.5)	130-262 (M=177.8)	113-199 (M=147.6)	112-340 (M=168.7)	98-325 (M=203.6)	94.5-251 (M=158.5)
HDL mg/dl	35-53 (M=42.4)	30-51 (M=42.6)	29-65 (M=44.7)	27-58 (M=40)	26-57 (M=42)	27-55 (M=36)	24-67 (M=42.6)	18-63 (M=42)	18-58 (M=39.6)
LDL mg/dl	43.8-141 (M=100.7)	85-180 (M=118.9)	58.6-142.7 (M=90.7)	77-176 (M=112.8)	79-195 (M=120.7)	58.6-142.7 (M=93.5)	51.4-249.6 (M= 106.8)	41-324 (M=137.2)	48.2-183 (M=95.3)
Chol/HDL	2-5.2 (M=3.84)	3-6 (M=4.51)	2.8-5.7 (M=3.71)	2.7-7 (M=4.4)	3.6-7 (M= 4.67)	3-6.8 (M=4.11)	2.1-10 (M=4.08)	2.5-10.5 (M=5.29)	2.45-7.57 (M=4.16)
Apo A1	1.11-1.29 (M=1.28)	0.94-1.62 (M=1.36)	1.11-1.89 (M=1.36)	0.99-1.61 (M=1.21)	3.62-7.08 (M=4.68)	3-6.8 (M=4.11)	0.78-1.65 (M=1.24)	0.86-1.29 (M=1.1)	0.63-1.7 (M=1.23)
Apo B	0.28-0.99 (M=0.60)	0.84-1.11 (M=0.94)	0.66-1.26 (M=0.81)	0.62-1.25 (M=0.90)	0.62-1.37 (M=0.95)	0.47-1.04 (M=0.74)	0.41-1.05 (M=0.77)	0.67-1.16 (M=0.94)	0.6-1.4 (M=0.94)

The results of the paired-samples *t*-test showed that there were statistically significant differences in three blood lipid parameters, namely cholesterol, LDL, and cholesterol/HDL ratio in three groups between baseline, MFKD, and PUFA-KD. In group 1, the mean cholesterol level of patients on MFKD (M = 189.7, SD = 33.32613) decreased when it was changed to PUFA-KD (M = 159.8, SD = 41.45042) and the difference was statistically significant t(6) = 3.463, *p* = 0.018. The mean LDL level of patients on MFKD (M = 118.9, SD = 32.91127) also decreased when it was changed to PUFA-KD (M = 90.7, SD = 31.88318) and the difference was statistically significant t(6)= 6.506, *p* = 0.001. The mean cholesterol/HDL ratio level of patients on MFKD (M = 4.50, SD = 0.98259) decreased when it was changed to PUFA-KD (M = 3.71, SD = 1.17279) and the difference was statistically significant t(6) = 3.140, *p* = 0.026. In group 2, the mean cholesterol level of patients on MFKD (M = 177.8, SD = 40.87562) decreased when it was changed to PUFA-KD (M = 147.6, SD = 26.71566) and the difference was statistically significant t(6) = 4.316, *p* = 0.002. The mean LDL level of patients at baseline (M = 112.8, SD = 31.88046) increased on MFKD (M = 120.7, SD = 34.04283) but decreased when it was changed to PUFA-KD (M = 93.5, SD = 23.07684) and the difference was statistically significant t(6)= 2.274, *p* = 0.046. The mean cholesterol/HDL ratio level of patients on MFKD (M = 4.67, SD = 1.11197) decreased when it was changed to PUFA-KD (M = 4.11, SD = 1.06038) and the difference was statistically significant t(6) = 3.281, *p* = 0.008. In group 3, the mean cholesterol level of patients at baseline (M = 168.7, SD = 55.06299) significantly increased on MFKD (M = 203.6, SD = 66.16439) t(6) = -2.268, *p *= 0.039 but decreased when it was changed to PUFA-KD (M = 158.5, SD = 44.76303) and the difference was statistically significant t(6) = 4.729, *p* = 0.001. The mean LDL level of patients at baseline (M = 106.8, SD = 46.52904) significantly increased on MFKD (M = 137.2, SD = 55.81106) t(6) = -2.322, *p* = 0.035 but decreased when it was changed to PUFA-KD (M = 95.3, SD = 38.34693) and the difference was statistically significant t(6) = 3.481, *p* = 0.004. The mean cholesterol/HDL ratio level of patients on MFKD (M = 5.29, SD = 2.14234) decreased when it was changed to PUFA-KD (M = 4.16, SD = 1.25618) and the difference was statistically significant t(6)= 2.964, *p* = 0.011. There were no significant differences in other blood lipid parameters in patients in the three groups. However, we observed in one group 3 patient that as compared to baseline (M = 103 mg/dl), there was an increase in the mean triglyceride level on MFKD (M = 558.7 mg/dl) and further on PUFA-KD (M = 802.75 mg/dl), but it was later brought within the normal range. We investigated but could not find the cause for the spike in the triglyceride level.

## Discussion

To the best of our knowledge, this is the first study in which the efficacy of using naturally occurring PUFA-KD in epilepsy has been trialed.

In our retrospective study, the change to a PUFA-KD from MFKD resulted in a statistically significant reduction in seizure frequency. Those who already had more than 90% reduction while on MFKD did significantly better than those with less than 90% reduction. We used natural oils in which other PUFAs are also present instead of purified forms of DHA and EPA.

We found efficacy with lower doses of DHA (1.17 g/day to 4 g/day mean of 2.21 g/day) than the optimum dose mentioned by Taha et al [2]. Several studies mention conflicting ratios of omega-6 to omega-3 fatty acids which may be an important determinant of brain health. Increased amounts of omega-6 in the diet leads to the production of larger quantities of eicosanoid metabolic products from arachidonic acid (AA) [9-10]. An imbalance of omega-6 and omega-3 PUFAs in the peripheral blood causes an excess production of pro-inflammatory cytokines which causes increase in excitatory glutamate and mitochondrial dysfunction [11-12]. Omega-3 suppresses the production of cytokines and increases the production of GABA and mitochondrial biogenesis [2]. Omega‐6 and omega‐3 fatty acids are both known to compete for the desaturation enzymes. Both fatty acid desaturase 1 and fatty acid desaturase 2 prefer alpha-linolenic acid (ALA) to linoleic acid (LA). Increased LA intake interferes with the desaturation and elongation of ALA. Thus a low ratio of omega-6 to omega-3 fatty acids can inhibit the conversion of large amounts of LA [9-10]. The n6:n3 ratio recommended by Yoon et al in his study is 2.8:1, but in our study, it was a mean of 4:1 (Group 2) to a mean of 12.6:1 (Group 3) [13]. We had to use a higher ratio of n6:n3 since fish oil is expensive in India and most of our patients from lower socio-economic strata had financial constraints. All vegetarians because of religious constraints refused the fish oil while some non-vegetarians refused this due to its odour.

There was no statistically significant difference in the n6:n3 ratio in the responders versus the non-responders. DHA did not seem essential as patients on n-6 PUFAs only (Group 1) and on n-6 PUFAs and n-3 PUFAs but without DHA (Group 3) also had good seizure control, though the number of the patients in the groups was too small to allow statistical analysis. The use of DHA and EPA is difficult due to our predominantly vegetarian population and also because of financial difficulties.

No serious side effects were seen in any of our patients. There was no significant abnormality in the blood lipid profile or other biochemical parameters in most patients. It was found that the mean cholesterol, LDL and cholesterol/HDL ratio levels of patients improved when the diet was changed to PUFA-KD as compared to baseline and while on MFKD. Tolerability of PUFA-KD was good and all those who left the diet did so due to an inadequate response, the restrictiveness of the diet and/or due to unacceptability of the taste of the fish oil.

Clinical trials on PUFAs supplementation in epilepsy have provided conflicting results [5-8, 14-15]. All these studies have evaluated the supplementation with omega-3 fatty acids, namely, purified forms of DHA and EPA. A recent Cochrane review concluded that in view of the limited number of studies and small sample sizes, there is not enough evidence to support the use of PUFAs supplementation in people with drug-resistant epilepsy [16]. There is limited data on the use of combined use of PUFAs with KD. Dahlin et al reported the effects on plasma fatty acids with omega 3 PUFAs supplementation in children on KD [17]. These children were supplemented with omega-3 fatty acids one month after the start of KD [17]. LA and EPA were increased, whereas AA and mead acid (20:3 n-6: n-9) were decreased [17]. DHA increased insignificantly. However, no correlation of changes in fatty acid levels with seizure response was found [17]. Yoon et al reported a case of a 7-year-old boy with Lennox-Gastaut syndrome combined with mitochondrial respiratory chain complex I deficiency, whose medically intractable seizures were successfully controlled with a PUFAs-enriched modified Atkins diet without any significant adverse events [13]. The diet consisted of canola oil and diverse menu items such as fish and nuts instead of olive oil and had a 1:2.8 ratio of omega-3 to omega-6 [13]. Schlanger et al conducted an open-label study where a fish oil spread was administered orally to provide 3.25g per day of n‐3 PUFAs (5g of the spread contained 46% DHA, 18% EPA, and 1% ALA). 16 patients out of 21 patients discontinued the study because of the “fishy” taste of the spread [5]. The five patients who completed the study, showed a significant reduction in seizure frequency (3 patients- 100 % seizure control and 2 patients- 78-99 % seizure control) after 6 months [5].

In the above studies, the amount of DHA used has been low (less than 3 grams/day) and the duration of the trials has been short. Taha et al. have suggested that DHA should be used in higher quantities (3-6 gm/day) and for longer periods (3-6 months) to improve seizure control [2]. This could be because oral DHA is trapped in the plasma chylomicron/LDL (low‐density lipoprotein) pools and does not increase in free form until a few weeks later when that pool is finally saturated. DHA levels in the brain may be slow to increase even after unesterified DHA is detected in the plasma because the half‐life of DHA in the human brain is around 2.5 years [2].

Evidence from in vitro, in vivo and human studies supports the concept that PUFAs have anti-epileptic effects [2]. PUFAs regulate the structure and function of neuronal membranes (raft and membrane-incorporated proteins) by maintaining the synaptic plasticity and fluidity of the lipid bilayer [18]. During epileptogenesis, the brain undergoes numerous changes including synaptic rearrangement and axonal sprouting, which require structural plasticity [19]. Fluidity and other parameters together with a hydrophobic thickness of bilayer have a prominent role in regulating many membrane functions, such as signal transduction, solute transport, and enzyme activity, associated with membranes [19]. Any alteration in lipid content due to lipid peroxidation leads to an imbalance in the distribution of inner and outer leaflets of the bilayer (asymmetry) and a decrease in fluidity [19]. Increased oxidative stress and changes in antioxidant capacity leading to membrane lipid peroxidation by mitochondrial dysfunction and release of excitatory neurotransmitters are considered important in the pathogenesis of epileptic seizures [19]. There are differences in the rate of mitochondrial beta-oxidation depending on the structure of fatty acids [20]. The oxidation of ALA is twice that of LA or oleic acid [20]. Therefore, ALA and LA are the most ketogenic of fatty acids and enriching a high-fat diet with these increases its ketogenic potential [20]. In vitro studies with n-3 PUFAs with ALA, EPA and DHA suggest several modes of action such as inhibitory effect on voltage-dependent sodium channels using DHA and stabilization of neuronal membranes by suppressing voltage-gated Na+ and Ca2+ channels leading to an increase in action potential firing threshold and overall reduction in bursting in the hippocampus and neuronal excitability [21-24]. PUFAs may activate a lipid‐sensitive class of K2P potassium channels and also enhance the activity of the Na+/K+‐ATPase (sodium pump) [25]. PUFAs might indirectly induce the expression and activity of uncoupling proteins (UCPs) to diminish reactive oxygen species (ROS), reduce neuronal dysfunction and induce a neuroprotective effect [25]. PUFAs also activate peroxisome proliferator-activated receptors (PPARα) that inhibit pro-inflammatory transcription factors thereby reducing inflammation which is a core contributor to seizures [25]. PPARα also induces a coordinated up‐regulation of energy transcripts leading to enhanced energy reserves and limited hyperexcitability [25]. Studies show that PUFAs also reduce oxidative stress by decreased activity of pro-apoptotic factors and inhibition of inflammatory mediators such as interleukins and tumor necrosis factor-alpha (TNFα) [25]. EPA reduces inflammatory mediators by inhibiting phosphokinase A2 and cyclooxygenase-2 enzymes metabolized to anti-inflammatory series 3 prostaglandins, which is implicated in epilepsy [26]. In vivo studies also have provided proof of anticonvulsant efficacy of PUFAs [27-28]. Studies with n-6 PUFAs are few but AA has been found to inhibit sodium ion channels [29]. There have been studies that provide evidence that a lower dose of EPA actually elevates membrane AA phospholipid concentrations in the neuronal membrane which is important for health in terms of cognition, longevity and cardiovascular health [30]. It was observed that EPA administration produced clinical improvement in schizophrenia patients, but that improvement was greater at a dose of 2 g/day than at 4 g/day [30].

However, there are several drawbacks of this trial, including the retrospective nature of the trial and the lack of one standard intervention. This is because the caregivers and patients often refused certain oils due to religious beliefs, and/or unpleasant taste. In spite of these, this trial challenges certain concepts and gives a new form of KD which may prove superior to the classical KD.

## Conclusions

PUFAs can be an important component of ketogenic diet to improve the efficacy of KD in those who have not achieved satisfactory control in seizures or have reached a plateau on MFKD. We found that both n-6 PUFAs and n-3 PUFAs rich oils were effective for seizure control. In future, randomized controlled trials will be needed to compare the efficacy of MFKD versus PUFA-KD and determine the ideal ratio of n-6 PUFAs to n-3 PUFAs as well as the total amount of DHA required. The PUFA-KD overall had good compliance, tolerability, and few side-effects. Cholesterol, LDL and cholesterol/HDL ratio levels significantly improved in patients on PUFA-KD as compared to baseline and while on MFKD.
